# 3D mesenteric angiogram-based assessment of Arc of Riolan crossing the inferior mesenteric vein: important considerations in high ligation during splenic flexure takedown in anterior resection

**DOI:** 10.1007/s00276-022-02992-x

**Published:** 2022-07-23

**Authors:** James Wei Tatt Toh, Geetha Ramaswami, Kim Son Nguyen, Geoffrey Peter Collins, Edgardo Solis, Nimalan Pathma-Nathan, Toufic El-Khoury, Seon Hahn Kim

**Affiliations:** 1 Discipline of Surgery, Sydney Medical School, The University of Sydney, Sydney, Australia; 2 Colorectal Department, Westmead Hospital, Sydney, Australia; 3 Department of Radiology, Westmead Hospital, Sydney, Australia; 4 The University of Notre Dame, Sydney, Australia; 5 Colorectal Department, Korea University Anam Hospital, Seoul, South Korea; 6 Korea University College of Medicine, Seoul, South Korea; 7 Colorectal Department, Division of Surgery and Anaesthetics, Westmead Hospital, Westmead, Cnr Hawkesbury and Darcy Rd, Sydney, 2145 Australia

**Keywords:** Arc of Riolan, Intermesenteric trunk, Meandering mesenteric artery, Colorectal surgery, Anatomy, Operative technique

## Abstract

**Background:**

Recent studies have described the finding of the Arc of Riolan (AoR) crossing the inferior mesenteric vein (IMV) seen during high ligation of IMV while performing minimally invasive colectomies. However, the AoR usually has a medial course, and this variant AoR anatomic course and the clinical importance of its preservation during splenic flexure takedown in anterior resection remains controversial.

**Methods:**

After institutional approval (QA-5775), radiological identification of and mapping of the vessel horizontally crossing the IMV under the pancreas, when present, was performed at a single institution (Westmead Hospital, New South Wales, Australia). One hundred consecutive computed tomographic (CT) mesenteric angiograms conducted in 2018 were reviewed retrospectively to determine the presence of a vessel horizontally crossing the IMV. 3D reconstructions were used to map out its course to understand its origin and full course. Baseline characteristics, including demographic and comorbidity data, were obtained from the medical record.

**Results:**

On 3D mesenteric angiogram reconstructions, a vessel crossing anterior to the IMV was present in 11 of 98 cases (11.2%). Two cases were excluded as the presence of this vessel was indeterminate. Eight of 11 patients (72.7%) were male, and the mean age was 49.3 years (range: 21–80 years). There was no statistically significant difference in age and comorbidities between the groups. Importantly, in all 11 cases, there was an arterial vessel crossing the IMV originating from the SMA and communicating with the IMA or a branch of the IMA, proving definitively that this vessel was by definition the AoR.

**Conclusion:**

This 3D mesenteric angiogram mapping study has shown definitively that the vessel horizontally crossing anterior to the IMV and inferior to the pancreas is an arterial vessel from the SMA to IMA, and by definition the Arc of Riolan. When present, identification and preservation of this collateral arterial vessel during splenic flexure takedown in anterior resection may be important in reducing the risk of post-operative bowel ischaemia.

## Introduction

Recent studies have described a vessel which crosses the IMV just below the inferior border of the pancreas. In a retrospective review of 1299 operative videos of left-sided colonic resections, a vessel running horizontally below the inferior margin of the pancreas and crossing anterior to the inferior mesenteric vein (IMV) was observed in 17.8% of patients undergoing left-sided colectomy [16]. The study also demonstrated the importance of its preservation to reduce the risk of colonic and anastomotic ischaemia [16].

Furthermore, in experimental studies, this vessel has been temporarily occluded with a vascular clamp and indocyanine green (ICG) to demonstrate the importance of this vessel in maintaining adequate blood supply to the colon [13].

In these studies, while the vessel was postulated to be a variant of the Arc of Riolan, it was not possible to visualise the origin and full course of the vessel during surgery to confirm that the vessel flowed between the SMA and IMA, and as such, the minimally invasive surgical studies were not able to definitively demonstrate that the vessel crossing the IMV was the AoR.

The Arc of Riolan (AoR) is by definition a connection between the SMA and IMA but is distinguished from the marginal artery of Drummond by its more central location [6]. It most commonly joins the left branch of the middle colic and the ascending branch of the left colic arteries [7]. The reported incidence in the literature is less than 18% [4, 7, 10, 15, 17]. Although the vessel carries Riolan’s name, it was only vaguely described in his written work and was more thoroughly described by von Haller in 1786 [18]. In the presence of severe occlusive or stenotic disease of the SMA or IMA, this artery may be very dilated or tortuous and easily visible on catheter angiogram [12].

The collateral flow between the superior and inferior mesenteric arterial systems is extremely variable: a study of 400 cadavers from 1963 found no 2 identical specimens [11]. Despite its variability, its course is usually depicted as running medially and centrally from SMA to IMA. As well as the inconstant and variable AoR, a more constant collateral vessel is located more peripherally in the mesocolon. The marginal artery of Drummond is located in the mesocolon adjacent to the colonic wall, running the entire length of the colon, and is composed of branches from the right, middle and left colic arteries [4]. It is thought to be relatively deficient and sometimes absent at the splenic flexure, or Griffith’s point, where it is vulnerable to ischaemic insult at the watershed area between vascular territories [9]. In patients with a deficient marginal artery of Drummond at the splenic flexure, the AoR provides critical collateral flow important to prevent ischaemia.

Herein lies the dilemma. Commonly cited reference texts and review articles such as those by Corman, Sabiston, Last and Gourley et al. [3, 6, 8, 14] have depicted the AoR as a vessel with a medial, vertically oriented course rather than a vessel with a horizontal course traversing the IMV. However, recent surgical studies during minimally invasive surgery have described an important collateral arterial vessel horizontally crossing the IMV and have postulated it to be the AoR, but without being able to trace its full course or origin during surgery.

The AoR is an important collateral vessel but it is inconstant and variable. While its importance in providing collateral flow is well known, until recently, its relation to the IMV was not well described. Thus, to definitively confirm that the vessel crossing the IMV horizontally just under the pancreas was the AoR, radiological mapping of 100 consecutive CT mesenteric angiograms (CTAs) was performed.

## Methods

Institutional approval was obtained for this study (QA-5775). Data were retrospectively collected for 100 consecutive patients undergoing computed tomography (CT) angiogram studies in 2018 for any indication at Westmead Hospital, NSW, Australia. CT mesenteric angiograms were obtained 20–25 s following the injection of intravenous contrast.

CT specifications: Toshiba (Canon) Aquilion Prime, 0.5 × 80 detector rows, 0.35 s rotation time; Siemens Somatom Force, dual source dual energy CT, 2 × 192 × 0.6 detector rows, 0.25 s rotation time; Collimation-40 cm. Images were obtained via volumetric core dataset acquisition with 1 mm thick slices and 3 mm thick multiplanar reformats.

The mesenteric arterial anatomy was defined using two independent reads by two consultant radiologists. The images were also reviewed by a specialist colorectal surgeon. The three specialists assessed the 100 CT mesenteric angiograms to examine the presence of a vessel crossing the IMV under the pancreas. When present, the origin and full course of this vessel was carefully traced to determine if it was the AoR. Image interpretation was aided by three-dimensional (3D) reconstruction, and 3D mesenteric angiogram assessment was used to definitively trace the vessel.

The following criteria were used to identify the vessel as the AoR: a vessel joining the SMA or a branch thereof, to the IMA, left colic artery or ascending branch of left colic artery. The marginal artery of Drummond must be identified as a separate collateral vessel.

Baseline demographic and comorbidity data were obtained from the medical record for each patient. Statistical analysis was conducted using Stata/MP, version 15 (StataCorp LP, College Station, TX) and *χ*^*2*^ Fisher’s exact test.

## Results

Of the 100 consecutive patients identified who underwent CT angiogram, two were excluded as the presence of the vessel crossing the IMV was unable to be determined. Of the remaining 98 cases, a vessel crossing the IMV under the pancreas was found to be present in 11 patients (11.2%).

In each of these cases, the vessel ran horizontally along the inferior border of the pancreas and crossed anterior to the IMV. The vessel originated from the SMA and in all 11 cases and joined the ascending branch of the left colic artery. By definition, in all cases, the vessel horizontally crossing the IMV under the pancreas was the AoR.

**Fig. 1:**
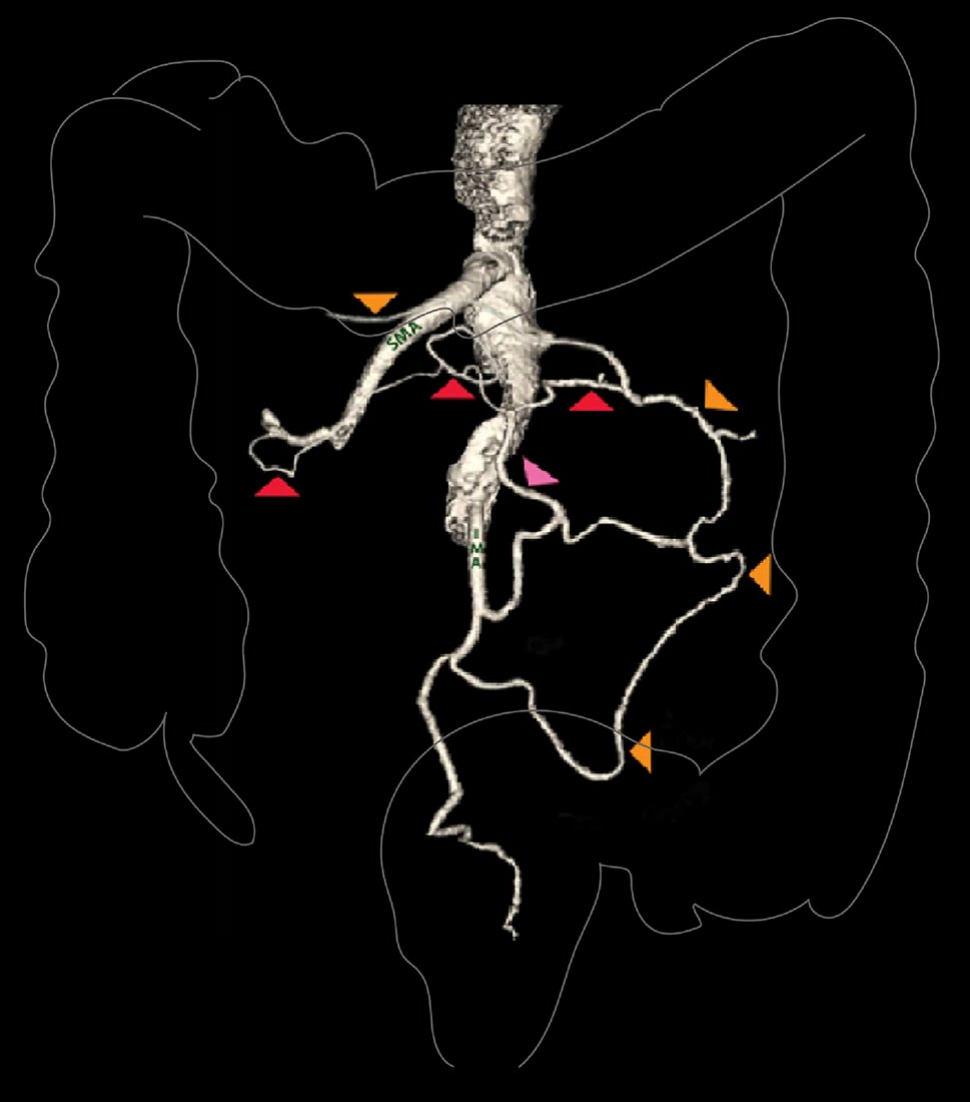
3D CT mesenteric angiogram. AoR (red arrow) joining the middle colic artery to the ascending branch of left colic artery (pink arrow). The marginal artery of Drummond is seen as a separate and distinct vessel (orange arrow)

**Fig. 2:**
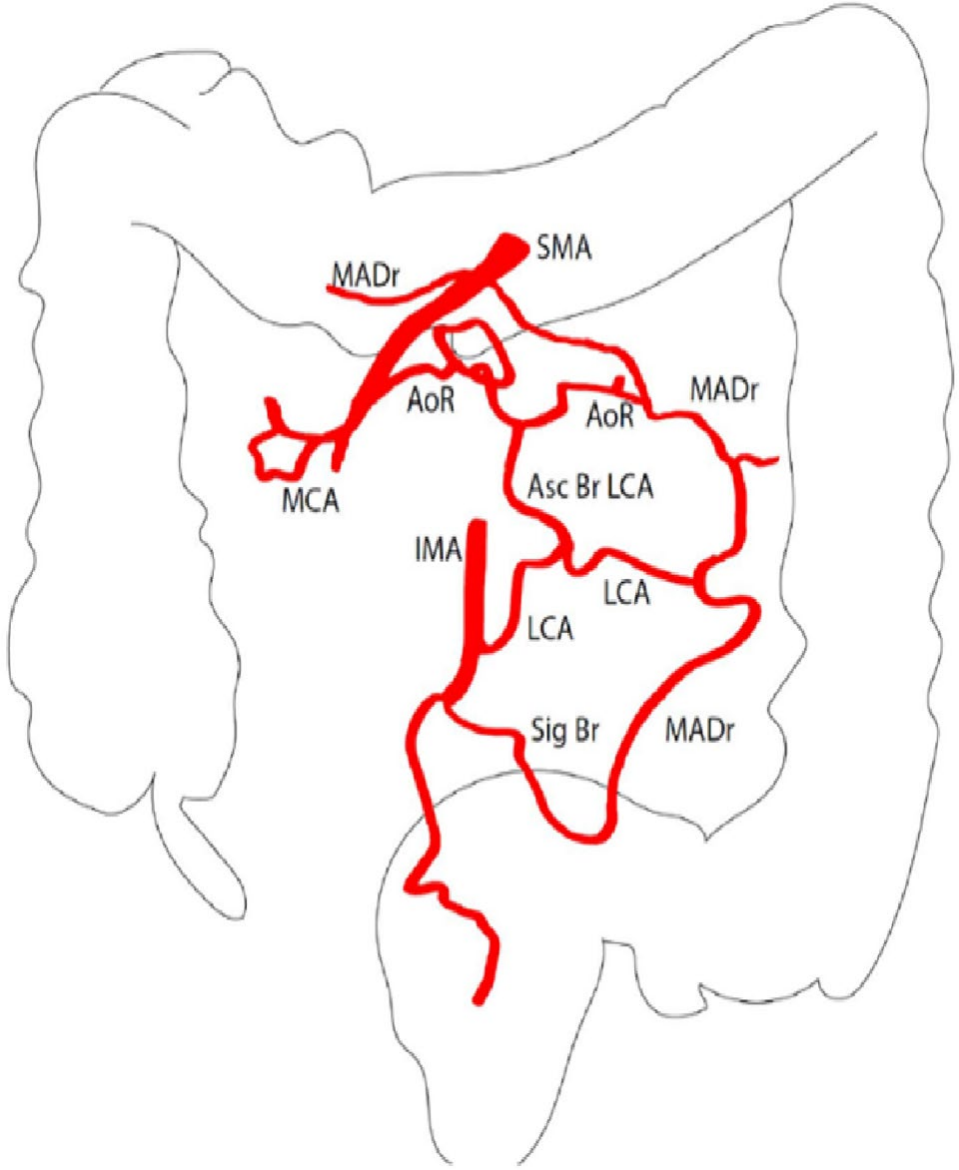
Schematic representation of 3D reconstruction. AoR: arc of Riolan, MADr: marginal artery of Drummond, MCA: middle colic artery, SMA: superior mesenteric artery, IMA: inferior mesenteric artery, Asc Br LCA: ascending branch of left colic artery, LCA: left colic artery, Sig Br: sigmoid branches

A representative 3D reconstruction and accompanying schematic labelled representation has been provided in Figs. 1 and 2, depicting the origin and full course of the horizontal vessel crossing the IMV which has been demonstrated as a collateral arterial vessel that joins the SMA to IMA.

Indications for the CT angiogram studies included in this study included major trauma, chest pain and operative planning for cardiothoracic or vascular surgery. There was no statistically significant difference in age, gender or comorbidity between the groups. Patient demographic and comorbidity data have been summarised in Table 1.

**Table 1:** Presence of AoR and association with baseline patient demographics and comorbidities. Values presented as Odds Ratio (OR) with 95% confidence interval (95% CI) with *P* values (*χ*^*2*^ Fisher’s exact test)

	OR	95% CI	P value
Age > 70	1.96	0.30–9.47	0.29
Male	0.94	0.20–6.00	1.00
AAA	n/a	–	–
IHD	0.63	0.01–5.20	1.00
HTN	1.58	0.35–6.78	0.52
High cholesterol	0.99	0.09–5.47	1.00
Vascular disease	1.35	0.03–13.06	0.58
Malignancy	n/a	–	–

## Discussion

Much of the surgical literature relating to the AoR focuses on the nomenclature and anatomic variation, with less emphasis on regional operative considerations, particularly with respect to the IMV.

Our 3D mesenteric angiogram mapping study clearly demonstrates that the vessel horizontally crossing the IMV under the pancreas which is at risk of injury during high ligation of the IMV was, by definition, the AoR. This confirms the anatomy of the AoR described in the previous study of 1299 operative cases [16].

Prior to this study, the traditional view was that the AoR took a more medial, vertical course [3, 6, 8, 14]. The limitation of previous operative studies was that only part of the course of the horizontal vessel was visualised and its origin and full course was not definitively traced as the AoR. Radiological mapping in this study was able to confirm that the horizontal vessel crossing the IMV was the AoR.

There have been a few detailed descriptions in the literature of operative considerations relating to patients with an AoR undergoing colorectal surgery. In a previous study [16], the AoR was identified in 17.8% of patients, consistent with the incidence reported in the literature. This study builds on those findings, using CT angiography to confirm that the vessel running inferior to the body of the pancreas and crossing the IMV horizontally was definitively the AoR as it connected the SMA and IMA territories. Division of this vessel in the presence of a deficient marginal artery of Drummond may increase the risk of post-operative colonic ischaemia due to inadequate collateral flow from the SMA.

Prior to these studies, Al-Asari et al. (2013) reported on a small case series of 30 consecutive patients undergoing minimally invasive colorectal surgery assessing for collateral vessels during dissection and ligation of the IMV. In this study, 13 of 30 patients (43.3%) were reported as having an AoR crossing the IMV, a much higher incidence than what has been reported in the literature [1]. The study emphasised on the importance of skeletonising the IMV to avoid injury to nearby collateral vessels.

In a systematic review, Bruzzi el al. (2019) suggested that division of the AoR (which was referred to as the intermesenteric trunk [IMT]) may cause colonic ischaemia in the presence of a discontinuous marginal artery of Drummond [2]. In this study, there was no direct reference to regional anatomical considerations, but the study suggested that preservation of this IMT makes obtaining adequate colonic length after IMV ligation and splenic flexure mobilisation more difficult.

Garcia-Granero et al. (2017) conducted a study on 27 human cadavers, performing a laparoscopic medial approach to splenic flexure mobilisation [5]. In this study, a central communication between the middle colic and the ascending branch of the left colic arteries (referred to as the artery of Moskowitz) was found in 3 of 27 cadavers (11%). The study reported potential difficulty with the medial approach in fully mobilising the splenic flexure when there is an artery of Moskowitz. However, in this study, there was no mention of its relationship with the IMV.

Unfortunately, the nomenclature in describing collateral arterial flow supplying the colon is inconsistent and confusing. Various names have been used in the literature, including but not limited to meandering mesenteric artery, mesomesenteric artery, IMT, middle-left colic collateral, arch of Treves, artery of Moskovitch, artery of Gonzalez and anastomosis maxima of Haller. To add to the confusion, some of these are considered by some authors to be separate entities [7]. For example, some consider the AoR and the artery of Moskovitch to be anatomically distinct vessels [5].

In addition, in a systematic review from 2019 [2], Bruzzi et al. described the AoR as connecting the distal left branch of the middle colic artery to the ascending branch of the left colic artery, running parallel to the marginal artery of Drummond in the peripheral mesocolon. The study separately termed a more central connecting vessel in the region of the inferior border of the pancreas and the inferior IMV the IMT.

Regardless of the terminology and definitions used, when present, the centrally located arterial connection between the SMA and IMA territories may have significant implications for left-sided colorectal surgery. While the name and course of the AoR is confusing, this study has definitively demonstrated that the vessel crossing the IMV under the inferior border of the pancreas is the AoR.

## Limitations

**Fig. 3:**
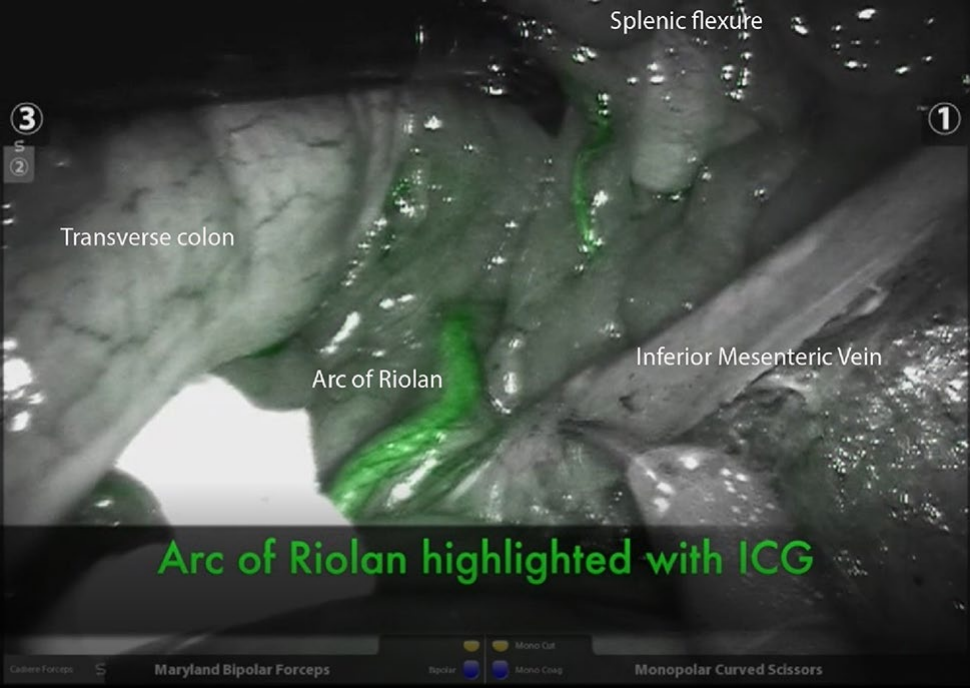
Indocyanine green Firefly fluorescence imaging on Da Vinci^™^ to aid in visualisation of the AoR intraoperatively

Mesenteric CT angiograms are associated with more radiation than standard CT and the purpose of this study was not to recommend 3D mesenteric angiograms to be performed to assess colonic vasculature prior to colonic surgery. In this study, mesenteric angiograms were performed mainly for trauma and vasculature diseases rather than to assess colonic vasculature prior to colonic surgery. Importantly, the aim of the study was not to show the value of 3D mesenteric angiograms in planning for colonic surgery. Rather, the aim was to definitively confirm that the collateral vessel crossing the IMV under the pancreas was arterial, and importantly, the AoR, which we have achieved. This is not possible using standard CT and it is not clinically appropriate to dissect out the course of this vessel to its SMA origin due to the significant risk of bleeding. Thus, this mapping study is an important study to provide clarification on the anatomy of the collateral vessel traversing the IMV under the pancreas. Intraoperatively, use of newer technologies without significant radiation dose such as indocyanine green Firefly fluorescence imaging on Da Vinci^™^ (Fig. 3) may aid in visualisation of the AoR, and may help in sparing the AoR during high ligation of IMV while performing splenic flexure takedown in anterior resection.

## Conclusion

This 3D mesenteric angiogram mapping study has confirmed that the vessel horizontally crossing anterior to the IMV and inferior to the body of the pancreas which is at risk of injury during high ligation of the IMV is the AoR, a collateral vessel between the SMA and IMA. Its identification and preservation during left-sided colectomies by performing an AoR sparing splenic flexure takedown may reduce the risk of post-operative bowel and anastomotic ischaemia by preserving collateral vessel arterial supply.

## References

[1] Al-Asari SF, Lim D, Min BS, Kim NK (2013). The relation between inferior mesenteric vein ligation and collateral vessels to splenic flexure: anatomical landmarks, technical precautions and clinical significance. Yonsei Med J.

[2] Bruzzi M, M'Harzi L, El Batti S, Ghazaleh RA, Taieb J, Poghosyan T (2019). Inter-mesenteric connections between the superior and inferior mesenteric arteries for left colonic vascularization: implications for colorectal surgery. Surg Radiol Anat.

[3] Corman ML, Corman ML (2005). Colon and rectal surgery.

[4] Drummond H (1913). The arterial supply of the rectum and pelvic colon. Brit J Surg.

[5] Garcia-Granero A, Sánchez-Guillén L, Carreño O, Sancho Muriel J, Alvarez Sarrado E, Fletcher Sanfeliu D (2017). Importance of the Moskowitz artery in the laparoscopic medial approach to splenic flexure mobilization: a cadaveric study. Tech Coloproctol.

[6] Gourley EJ, Gering SA (2005). The meandering mesenteric artery: a historic review and surgical implications. Dis Colon Rectum.

[7] Lange JF, Komen N, Akkerman G, Nout E, Horstmanshoff H, Schlesinger F (2007). Riolan’s arch: confusing, misnomer, and obsolete. A literature survey of the connection (s) between the superior and inferior mesenteric arteries. Am J Surg.

[8] Last R (1956). Anatomy: regional and applied.

[9] Meyers M (1976). Griffiths’ point: critical anastomosis at the splenic flexure. Significance in ischemia of the colon. AJR Am J Roentgenol.

[10] Michels NA (1963). The variant blood supply to the small and large intestines: its import in regional resections. J Int Coll Surg.

[11] Michels NA, Siddharth P, Kornblith PL, Parke WW (1965). The variant blood supply to the descending colon, rectosigmoid and rectum based on 400 dissections. Its importance in regional resections. Dis Colon Rectum.

[12] Moskowitz M, Zimmerman H, Felson B (1964). The meandering mesenteric artery of the colon. AJR Am J Roentgenol.

[13] Park H, Piozzi GN, Lee TH, Kim JS, Choi HB, Kim SH (2021). Arc of Riolan-dominant colonic perfusion identified by indocyanine green after high ligation of inferior mesenteric artery: critical in preventing anastomotic ischemia. Dis Colon Rectum.

[14] Sabiston DC, Townsend CM, Beauchamp R, Evers B, Mattox K (2001). Sabiston textbook of surgery: the biological basis of modern surgical practice.

[15] Steward J, Rankin FW (1933). Blood supply of the large intestine: its surgical considerations. Arch Surg.

[16] Toh JWT, Matthews R, Kim S-H (2018). Arc of Riolan-preserving splenic flexure takedown during anterior resection: potentially critical to prevent acute anastomotic ischemia. Dis Colon Rectum.

[17] VanDamme J (1993). Behavioral anatomy of the abdominal arteries. Surg Clin North Am.

[18] von Haller A, Cullen W, King LS (1966). First Lines of Physiology: Translated from the Correct Latin Edition, Printed Under the Inspection of William Cullen, and Compared with the Edition Published by HA Wrisbert: a Reprint of the 1786 Edition, with New Introd.

